# *Suan Zao Ren Tang* as an Original Treatment for Sleep Difficulty in Climacteric Women: A Prospective Clinical Observation

**DOI:** 10.1155/2011/673813

**Published:** 2011-05-10

**Authors:** Chia-Hao Yeh, Christof K. Arnold, Yen-Hui Chen, Jung-Nein Lai

**Affiliations:** ^1^Institute of Traditional Medicine, School of Medicine, National Yang-Ming University, Taipei 112, Taiwan; ^2^Department of Chinese Medicine, Taipei City Hospital, Yang Ming Branch, Taipei 112, Taiwan; ^3^School of Pharmacy, College of Medicine, National Taiwan University, Taipei 100, Taiwan; ^4^Department of Obstetric and Gynecology, Taipei City Hospital, Yang Ming Branch, Taipei 112, Taiwan

## Abstract

Little scientific evidence supports the efficacy of herbal medicines in the treatment of women with sleep difficulty during the climacteric period. The purpose of this study is to evaluate the efficacy and safety of *Suan Zao Ren Tang (SZRT)* in reducing the impact of sleep disturbance on climacteric women, as measured by Pittsburg sleep quality index (PSQI) and the World Health Organization quality of life (WHOQOL). Sixty-seven climacteric women with sleep difficulty intending to treat received *SZRT* at a rate of 4.0 g, thrice daily for four weeks (MRS < 16, n = 34; MRS ≥ 16, n = 33). After taking into account potential confounding factors, the mean PSQI total scores had fallen from 13.0 (±2.9) to 9.0 (±3.2) (95% confidence interval −4.93, −3.10). Further analyses showed that *SZRT* produced superior benefit of daytime dysfunction in women with severe menopausal symptoms (MRS ≥ 16). There were three of the withdrawals involved treatment-related adverse events (stomachache, diarrhea, and dizziness). Excluding women with a past history of stomachache, diarrhea, or dizziness, four weeks of therapy with *SZRT* appears to be a relatively safe and effective short-term therapeutic option in improving daytime function of climacteric women with poor sleep quality.

## 1. Introduction

Insomnia is not only one of the most common symptoms of menopause [1–4], but also one of the major determinants relating to quality of life (QOL) for climacteric women seeking medical advice [5]. This condition occurs in over 50% of climacteric women and could act, in an independent or synergistic way, with various menopausal symptoms to impact their daytime function [5, 6]. For treating insomnia, benzodiazepines quickly gained acceptance as the preferred treatment after a debut on the pharmaceutical market in 1960. Despite clinical guidelines recommending their use [7], there are still claims that the benefits of taking this older generation of hypnotics are outweighed by serious risks including daytime sedation, dependence, rebound insomnia, slurred speech, staggering gait, poor judgment, and slow uncertain reflexes. Not surprisingly, many women with sleep difficulty have turned to traditional medicine in search of a way to manage their sleep problems and promote their QOL.

*Suan Zao Ren Tang *(*SZRT*) has a long history of use as part of the traditional Chinese pharmacopoeia first documented in the classical Chinese text *Jin Gui Yao Lue* (Essential Prescriptions from the Golden Cabinet) circa 210 A.D. by Zhong-Jing Zhang [8]. *SZRT* is a combination of five medicinal Chinese herbs as follows: *Semen Zizyphi Spinosae *(*Suanzaoren*),* Sclerotium Poriae Cocos *(*Fuling*),* Radix Ligustici Chuanxiong *(*Chuanxiong*),* Rhizoma Anemarrhena *(*Zhimu*), and *Radix Glycyrrhizae *(*Gancao*) [9] (Table 1). In the classical literature, *SZRT* is said to nourish the blood and calm the nerves to eventually bring on a tranquillizing sensation and reduce the effect of sleep disturbance. Traditional medicine (TM) practitioners in Japan, South Korea, China, and Taiwan, which have a similar history of traditional Chinese medicine (TCM), rely on the indications and contraindications mentioned in the thousand-year-old classical literature of Chinese medicine.

Our previous study demonstrated that *SZRT* is frequently prescribed for climacteric women in Taiwan [10, 11]. In the United States, *SZRT* is also available, but as an over-the-counter dietary supplement. Despite its popularity, *SZRT* does not have any scientific evidence to back up claims of efficacy or safety in clinical use. Pharmaceutical companies are generally reluctant to conduct any randomized controlled trial for *SZRT* that is already on the market and approved by regulatory bodies. However, it is unethical not to conduct clinical trials to outline the safety profile of *SZRT*; clinical trials will also provide basic knowledge to explore *SZRT*'s possible mechanisms of effectiveness. Therefore, an observation period of four weeks can start to expose any unknown side effects and may verify an ancient indication that *SZRT* is effective in improving sleep quality, sleep latency, sleep disturbance, sleep efficiency, and daytime function during the climacteric period, a complex stage in the life of a woman.

## 2. Methods

### 2.1. Herbal Formula

The *SZRT* formula used in this study is a herbal extract powder from the good manufacturing procedures (GMP) of the certified company Kaiser Pharmaceutical Co., Ltd. (Taiwan), for the purpose of meeting international market standards of quality and uniformity. The project was entirely funded by the Taipei Chinese Medical Association to detect potential toxicity or AEs of *SZRT*. Apart from providing the batch of* SZRT*, Kaiser Pharmaceutical was not involved in any other sponsorship, study design, or monitoring of the participants.

### 2.2. Preparation of the Herbs

All herbal components of *SZRT* were prepared in a large computer-controlled boiler where volatile oils and herbal broth were collected, pumped into a vacuum drying condenser, and sprayed onto a base material. These granulated compounds were then vacuum-dried at low temperatures before being siphoned into a separate sterile-room, where they were bottled, labeled, and sealed. Granules were packed in aluminum foil packages and administered orally at a dose of 4 g, three times per day. For quality assurance of the active ingredients in *SZRT*, HPLC (high performance liquid chromatography) fingerprinting was employed to identify substances in the final product. No animal products, endangered species, or restricted herbal ingredients were used in this study. Each batch of *SZRT* was also tested for *E. coli, Salmonella *(bacteria count), and heavy metals.

### 2.3. Recruitment of Subjects

The Yang Ming branch of the Taipei City Hospital was the location for patient enrollment in the study. The study obtained approval from the Taipei City Hospital Institutional Review Board (TCHIRB-970110-E). Participants were recruited through newspaper advertisements and flyers posted in clinics and health fairs between April 2008 and April 2010. All participants understood the nature of the trial and willingly signed the informed consent form.

### 2.4. Eligibility Criteria

The subjects enrolled in the study were climacteric women, ranging from 40–65 years of age, with a Pittsburg sleep quality index (PSQI) of greater than six. These women spent 45 minutes or more falling asleep, slept less than six hours, and had struggled with this issue continuously for at least one month.

### 2.5. Ineligibility Criteria

The reasons women were unable to participate in the study were as follows: (1) within three months of the study there had been a major incident, (2) working the nightshift, (3) lack of time or timing conflicts, (4) confounding diseases such as psychosis, severe depression, obstructive sleep apnea-hypopnea, restless legs syndrome, cardiac arrhythmia, acute myocardial infarction, high blood pressure, diabetes, or cancer, (5) currently taking pharmaceuticals that affect melatonin, acetylcholine, glutamate, serotonin, norepinephrine, GABA, histamine, adenosine, or prostaglandins, (6) inability to read and fill out the forms for the study, and (7) any evidence of renal or liver dysfunction as defined by a level of at least 1.5 times of the upper reference limit (serum creatinine: 1.3 mg/dL, blood urea nitrogen (BUN): 22 mg/dL, serum aspartate-aminotransferase (AST): 25 IU/L, alanine-aminotransferase (ALT): 29 IU/L).

### 2.6. Study Design and Procedure

Nurses were briefed on important items prior to the start of the study and attended a training session to ensure duties were conducted in accordance with good clinical practice (GCP) guidelines. Participants passed through an initial screening phase of two visits. On the first visit, they were interviewed to obtain information about medical history and were later subjected to a clinical examination. The second visit determined the baseline data on symptoms, quality of life, and a basic health assessment that consisted of complete blood counts and biochemical function tests. These tests were performed to ensure the eligibility of each woman and to identify respondents with potential poor compliance. At the end of the second visit, each eligible woman was provided with sufficient *SZRT* at a treatment dose of 4 g thrice daily to take until the next visit. And no other herbals were allowed during the study period.

Followup visits were scheduled for the first and fourth weeks of the study. Physical examinations, basic health assessments, and biochemical function tests for each woman were carried out at the end of the study. At the end of the trial, the participants received a further physical examination, including blood tests. Women were contacted by telephone one to two days prior to each visit to encourage their continued participation. Throughout the study, leftover package counts were done to monitor each woman's compliance. The occurrence of AEs was recorded on every visit.

### 2.7. Efficacy/Tolerability

The primary outcome parameter evaluated prior to and following the four week intervention was the Pittsburg sleep quality index (PSQI). The study included two secondary outcome parameters. The first was the World Health Organization quality of life questionnaire in the Taiwan brief version (WHOQOL-BREF). Number two, the menopause rating scale (MRS), consisting of 11 items, is rated on a five-point scale (0 = none, 4 = very severe) that permits description on the perceived severity of complaints such as hot flashes, sweating, sleep disturbance, and nervousness [12]. The questionnaire was developed to gain further insights into each woman's personal physiological changes and the psychological impacts commonly experienced by climacteric women [13]. The composite scores for each of the dimensions (subscales) are based on adding up the scores of the items of the respective dimensions. The composite score (total score) is the sum of the dimension scores. Useful categories for describing clinical relevance of the index were: total score ≥16 (severe complaints) and 1–15 (mild-to-moderate complaints).

The PSQI is a multidimensional, self-administered, health status measure, which probes clinically important, patient relevant symptoms in the areas of sleep quality and quantity. The PSQI comprises 19-item questionnaires with seven sub scales (subjective sleep quality, sleep latency, sleep duration, habitual sleep disturbances, use of sleep medication, and daytime dysfunction) [14]. Each subscale is rated 0–3 with the higher scores reflecting more severe sleep complaints. The sum of all the scores permits an analysis of the patient's overall sleep experience. The lower the overall score, the better the person sleeps. The tool possesses adequate internal reliability, validity, and consistency for clinical and community samples of the climacteric population [15]; however, the questions are heavily based on memory over the past month.

The Taiwan version of the WHOQOL-BREF comprises four domains (i.e., physical, psychological, social, and environmental) containing 24 facets, and two national items on overall quality of life (QOL) and general health [16]. The physical domain includes seven items (pain and discomfort, energy and fatigue, sleep and rest, mobility, daily living activities, dependence on medication, and working capacity), six in the psychological domain (positive feeling, thinking, and concentration, self-esteem, bodily image and appearance, negative feelings and spiritual/religious/personal beliefs), three in the social domain (personal relationships, social support, and sexual activity), and eight in the environmental domain (physical safety and security, home environment, financial resources, availability of health and social care, opportunities for acquiring new information and skills, participation in recreation and leisure, physical environment, and transport). Each item was scored on a Likert scale ranging from 1 to 5, with a higher score indicating a favorable condition. To standardize the domain scores for comparison, the average score of each domain was calculated and then multiplied by 4. Thus, the domain scores ranged from 4 to 20, with a higher score indicating a better quality of life in the corresponding domain.

### 2.8. Safety Assessment

To set up active safety surveillance, we first surveyed the TM practitioners currently using *SZRT* in the treatment of sleep difficulty to ascertain any AEs these practitioners had noticed. They reported that such medication occasionally resulted in gastrointestinal discomfort such as acid regurgitation, heartburn, and abdominal discomfort when *SZRT* was administered orally at a dose of 4 g, three times per day. We included these on our list for surveillance. We also included on our surveillance list the AEs most frequently reported by the National Reporting System: abdominal fullness, diarrhea, vomiting, nausea, urticaria, itching, purpura, jaundice, skin vesicle (or local reddish swelling), edema, hypotension, bradycardia, dyspnea, fever, muscle cramp, and sleepiness. Other possible AEs were enquired about as the 21st item. All subjects were required to take objective laboratory tests to detect any abnormal liver, blood, or kidney findings at the baseline and after four weeks of *SZRT* therapy.

### 2.9. Statistical Analysis

Our analysis focused mainly on changes in the seven-domain constructs of the PSQI assessment along with the WHOQOL measures. The study described the treatment effects as changes in mean differences among the participants between the baseline scores and those measured at visits on weeks one and four. Separate linear regression models were constructed for the response variables of differentiated scores on subjective sleep quality, sleep latency, sleep duration, habitual sleep disturbances, use of sleep medication, and daytime dysfunction subscales, and the WHOQOL-BREF domains. The explanatory variables in the final regression model were age, marital status, educational level, baseline severity of menopausal syndrome, and BMI (in kg/m^2^). To examine improvement across the month, two indicator variables for visits at weeks one and four were also included in the regression model. The coefficients of the two indicator variables represent the differences between the average value of measured scores for the visits at weeks one and four versus those at the baseline. We assumed correlated error terms among the repeated measurements for each participant. Finally, each single PSQI score item was also examined separately to identify any PSQI items sensitive to treatment by *SZRT*. All of the above analyses were performed using the SAS (9.2 edition) software package.

## 3. Results

### 3.1. Study Population

Of the 99 sample patients, 32 were deemed to be ineligible. The principal reasons for ineligibility were a disinterest in participation (*n* = 16), medical conditions and medications (*n* = 3), did not show up for further study (*n* = 11), and no evidence of severe sleep difficulty (*n* = 2). In all, 61 (91%) of the initial 67 participants intending to treat (ITT) completed the 4-week study without any major protocol violation. These were included in the per-protocol data set for safety and efficacy analyses. Reasons for the six6 withdrawals were AEs (*n* = 3), lack of efficacy (*n* = 1), and prior treatment of other disease (*n* = 2).

Table 2 summarizes the baseline demographics and clinical characteristics of the study. The women in the study exhibited a mean age of 55, normal BMI, 73% married, and 39% had an education level of junior high or below. To further separate out the confounding factors of multiple menopausal symptoms influencing the outcome of the study, participants were filtered into two groups consisting of mild-to-moderate menopausal symptoms (MRS < 16) versus severe menopausal symptoms (MRS ≥ 16), based on their baseline MRS score.

### 3.2. Safety and Adverse Effects

All tested biomarkers after the 4-week *SZRT *intervention did not demonstrate significant changes. However, the study raised notable safety issues. Three participants withdrew from the study due to AEs. Intolerable side effects resulting in withdrawal included three events of stomachache, diarrhea, and dizziness. We carried out periodic evaluation of any unexpected symptoms during the treatment period and found that the perception of gastrointestinal discomfort after *SZRT* consumption two days later and dramatically relieved after *SZRT* discontinuance. We were unable to rule out the possibility that consuming *SZRT* will cause the deterioration of gastrointestinal and/or neurologic symptoms preexisted during climacteric period. 

### 3.3. Efficacy

After four weeks of treatment, there were significant improvements in each of the PSQI subscales. The magnitudes of the improvements in the PSQI subscales were 37.5% for subjective sleep quality, 25% for sleep latency, 25% for sleep duration, 38.9% for habitual sleep disturbances, and 40% for daytime dysfunction (Table 3). The mean amount of time to fall asleep per day reduced from 53.6 ± 46.0 minutes at the baseline to 31.0 ± 20.0 minutes at the end of the study. The mean sleep duration per day prolonged from 4.7 ± 1.0 hours at the baseline to 5.5 ± 1.1 hours after 4-week treatment.

Among those intended to treat, 10 out of 67 women increased their MRS domain scores at the end of the study. Two of these women reported sleep quality at the same status as their baseline, one of these women reported sleep quality worse than her baseline, the remaining seven women perceived better sleep quality by week four.

The results of the multiple linear regression analyses indicate the effects of the different determinants on the outcome scores of the PSQI subscales. After controlling for the degree of severity of menopausal symptoms and other determinants had been controlled, the model constructions demonstrated significant improvements in the subjective sleep quality, sleep latency, sleep duration, sleep efficiency, sleep disturbances, and daytime dysfuction after 4-week treatment. In addition, the present data demonstrated that *SZRT* produced a superior benefit of daytime function in women with severe menopausal symptoms compared towith women with mild menopausal symptoms. However, there were no significant improvements in the domain scores of the WHOQOL-BREF after controlling for other potential confounders.

## 4. Discussion

To the best of our knowledge, this study is the first of its kind to investigate the potential efficacy and safety of *SZRT* in improving quality of sleep among climacteric women with sleep difficulty, as measured by PSQI. Our results demonstrate a significant improvement of sleep quality in climacteric women after four weeks of *SZRT* treatment. Although we used self-comparison to rule out potential confounding by BMI, smoking, exercise, and socioeconomic status, the possibility of a placebo effect might still exist. Thus, we further analyzed the improvement in subjective sleep quality, sleep latency, sleep disturbance, sleep duration, sleep efficiency, daytime dysfunction, and total PSQI scores by stratifying data into two groups with different degrees of severity of menopausal symptoms. A similar severity pattern in sleep difficulty and total PSQI scores were found at the baseline examination for these two groups (Table 2). Pronounced improvement of daytime dysfunction was found in the severe group after four weeks of *SZRT* treatment.

In the mild-to-moderate group, the mean total PSQI scores improved on average 3.6 points by the end of the study, and 73.5% of the participants perceived the improvement of sleep quality after four-weeks of *SZRT* treatment. 8.8% of the women with mild-to-moderate menopausal symptoms experienced deteriorated sleep quality over the study period, whereas 81.8% of the women with severe menopausal symptoms perceived the improvement of sleep quality at the end of the study. Nearly 12.1% of the women perceived poorer sleep quality after four weeks of *SZRT* treatment. Although head-to-head comparisons were not available, our results suggest that subjective sleep quality and daytime dysfunction of climacteric women with mild menopausal symptoms did not worsen during the treatment period. Although this study did not use a placebo group or randomization, the significant difference in improved score of daytime dysfunction indicated a positive effect of *SZRT* treatment in climacteric women who perceived poor sleep and severe menopausal symptoms (Table 4). This finding was similar to the findings of a prior study [17], in which sleep-related difficulty with daytime functioning significantly improved after a 4-week zolpidem treatment among women with menopause-related insomnia.

The present data showed that there was no effect after one-week *SZRT* treatment and was significant improvement for sleep quality after four-week treatment at a dose of 4.0 g thrice daily. From a physiologically based pharmacokinetic point of view, it takes more than one week to demonstrate the symptom-relief effect of the active ingredients in *SZRT*, which appears to resemble a slow-acting drug, and may not be appropriate to compare its action head-to-head with conventional medications of rapid action, such as zolpidem [17]. In the outcome evaluation study provides us precious evidence to avoid direct comparison of formula *SZRT* with rapid action medications in future randomized controlled trials.

Following the recommendations of the World Health Organization, we used the WHOQOL-BREF, a multidimensional measure of QOL, as one secondary outcome in this study [18]. However, there were no statistically significant changes in the scores of either the WHOQOL domains or the different facets for those climacteric women suffering from sleep difficulty who showed significant improvements in sleep quality after treatment (Table 5). A major reason for this result is that the mean scores of the WHOQOL-BREF at the baseline were comparatively high. Consequently, it may be unrealistic to expect a further marked increase in such scores. Another explanation was that because of its generic nature, the WHOQOL-BREF domain scores are not sensitive or responsive enough to detect changes.

Clinical trials have never explored the *SZRT *dosage used in this study (4.0 g thrice daily). Without the present data, TM practitioners in Japan, South Korea, China, and Taiwan will continue to prescribe *SZRT* to patients in their everyday practice based on documentations from a thousand years ago. The results of this study provide (about IIb level [18]) that three participants had to withdraw from the study due to AEs, and that the three leading adverse drug reactions included stomachache, diarrhea, and dizziness. The AEs discovered by this study, though not serious enough to prohibit the use of this formula clinically, show that TM practitioners must be alert to the possibility that prescribing *SZRT* can cause severe gastrointestinal adverse effects or dizziness. Further research to clarify the safety of *SZRT*'s use is necessary.

The present study recruited only climacteric women with poor sleep and used self-comparison to rule out the potential confounders of BMI, age, exercise, and socioeconomic status. This type of study presents particular advantages. Limited subject numbers create simple and comprehensive data analysis that can be used to identify possible trends, with which a larger sample size could prove to be significant. Participants do not have to spend the extended amount of time necessary for a crossover design, an alternate method for dealing with a limited number of subjects. Additionally, this type of study avoids the ethical dilemma regarding giving a placebo to symptomatic participants who could be receiving valid treatment.

A major limitation of our study is its observational nature or lack of a randomized placebo group. Therefore, our claim of an efficacy associated with *SZRT* might not be as strict as that from a RCT. No matter how popular the use of *SZRT* is in the markets of China, Japan, South Korea, and Taiwan, no RCT has been conducted yet. The evidence we obtained from the present study can provide precious data on safety profiles of *SZRT* and direct evaluation of its effectiveness, meanwhile this study can serve as prior information for the design of RCTs. Another limitation of this study is that since participants were all climacteric women with sleep problems in Taiwan, there needs to be caution in generalizing these findings to cover patients in other nations.

## 5. Conclusion

Excluding women with a past history of stomachache, diarrhea, and dizziness, the dosage form and prescription pattern of the *SZRT* preparation appears to be a well-tolerated and valuable, short-term alternative therapeutic option for improving daytime dysfunction in women with poor sleep quality during their climacteric period. Placebo effect, natural fluctuation, insufficient followup time, and/or other unobserved factors could confound the results of the current study. A prospective randomized, double-blind, controlled trial to further evaluate the efficacy of *SZRT* on climacteric women with sleep difficulty is warranted in future studies.

##  Conflict of Interests 

The author(s) declare that they have no Conflict of interests.

##  Authors' Contributions 

J.-N. Lai created the study design and coordination, patient recruitment, conceived the study, and helped draft the manuscript. C. K. Arnold conducted paper preparation and submission. J.-N. Lai and C. H. Yeh were responsible for data acquisition and obtained the funding for the study. C. H. Yeh collected all statistical analyses and interpretation of the data. All authors have read and approved the paper.

## Figures and Tables

**Table 1:** Nomenclature of Chinese herbs in *Suan Zao Ren Tang*.

Pharmaceutical name	Chinese Pinyin	Latin botanical name
*Semen Zizyphi Spinosae*	*Suanzaoren*	*Ziziphus jujuba var. Spinosa*
*Sclerotium Poriae Cocos*	*Fuling*	*Poria cocos (Schw.) Wolf*
*Radix Ligustici Chuanxiong*	*Chuanxiong*	*Ligusticum chuanxiong Hort.*
*Rhizoma Anemarrhena *	*Zhimu*	*Anemarrhena aspodeloidea Bunge*
*Radix Glycyrrhizae*	*Gancao*	*Glycyrrhiza uralensis Fisch.*

**Table 2:** Baseline demographic and clinical characteristics for the 67 participants.

Variables	MRS < 16 (*n* = 34)	MRS ≥ 16 (*n* = 33)	Odds ratio^†^
	mean (SD)	mean (SD)	
Demographics			
Age (year)	55.9 (6.8)	52.4 (6.0)	1.260*
Height (cm)	157.4 (6.7)	156.6 (4.7)	1.018
Weight (kg)	54.4 (7.3)	57.5 (11.0)	1.169
BMI (kg^2^/cm^2^)	22.6 (2.7)	23.4 (4.1)	0.551
Serum hormonal level			
FSH (mIU/mL)	50.4 (25.7)	51.9 (27.2)	0.948
LH (mIU/mL)	30.1 (16.1)	28.5 (13.8)	1.052
Endpoint (scores)			
PSQI	12.4 (3.3)	13.7 (2.1)	0.581
Subjective sleep quality	2.3 (0.5)	2.5 (0.5)	4.171
Sleep latency	2.4 (0.8)	2.4 (0.7)	2.652
Sleep duration	2.2 (0.8)	2.6 (0.6)	0.218*
Habitual sleep efficiency	1.7 (1.2)	2.0 (1.0)	1.559
Sleep disturbances	1.4 (0.6)	1.8 (0.6)	1.099
Daytime dysfunction	1.4 (0.9)	1.6 (1.0)	1.973
Domains of WHOQOL-BREF			
Physical	13.0 (2.1)	11.6 (1.7)	1.493
Psychological	12.3 (2.1)	10.4 (2.1)	2.185*
Social	13.3 (2.1)	12.5 (1.8)	0.818
Environmental	14.1 (1.8)	12.9 (2.2)	0.702

Abbreviation: BMI: body mass index; FSH: follicular stimulating hormone; LH: luteinizing hormone; MRS: the menopause rating scale; PSQI: the Pittsburg sleep quality index; WHOQOL-BREF: the World Health Organization's quality of life instrument—Short Version.

^†^Odds ratio are calculated for the comparison of difference between MRS < 16 and MRS ≥ 16 groups by logistic regression.

*P < .05

**Table 3:** Major variables of the measurements between weeks 1 and 4 and baseline are summarized as mean difference with a 95% confidence interval.

Variable definition	Baseline	Week 1	Paired *T*-Test	Week 4	Paired *T*-Test
	mean (SD)	mean (SD)	mean difference (95% CI)	mean (SD)	mean difference (95% CI)
Pittsburg sleep quality index	13.0 (2.9)	11.0 (3.4)	(−2.86, −1.26)^ ∗∗ ∗^	9.0 (3.2)	(−4.93, −3.10)^ ∗∗ ∗^
Subjective sleep quality	2.4 (0.5)	1.9 (0.6)	(−0.62, −0.27)^ ∗∗ ∗^	1.5 (0.7)	(−1.10, −0.69)^ ∗∗ ∗^
Sleep latency	2.4 (0.8)	2.1 (0.9)	(−0.44, −0.04)*	1.8 (1.0)	(−0.86, −0.36)^ ∗∗ ∗^
Sleep duration	2.4 (0.8)	2.1 (0.8)	(−0.44, −0.07)**	1.8 (0.8)	(−0.77, −0.36)^ ∗∗ ∗^
Habitual sleep efficiency	1.8 (1.1)	1.4 (1.1)	(−0.67, −0.16)**	1.1 (1.1)	(−1.04, −0.49)^ ∗∗ ∗^
Sleep disturbances	1.6 (0.6)	1.6 (1.3)	(−0.27, 0.39)	1.4 (0.6)	(−0.39, −0.03)*
Daytime dysfunction	1.5 (1.0)	1.1 (0.9)	(−0.67, −0.22)^ ∗∗ ∗^	0.9 (0.8)	(−0.88, −0.34)^ ∗∗ ∗^
WHOQOL-BREF Scores					
Physiological domain	12.3 (2.0)	13.0 (1.9)	(0.29, 1.05)^ ∗∗ ∗^	13.5 (2.3)	(0.64, 1.63)^ ∗∗ ∗^
Psychological domain	11.4 (2.3)	11.7 (2.6)	(0.00, 0.78)	11.9 (2.6)	(0.02, 0.96)*
Social domain	12.9 (2.0)	13.0 (2.1)	(−0.36, 0.48)	12.8 (2.5)	(−0.70, 0.43)
Environment domain	13.5 (2.1)	13.6 (2.2)	(−0.22, 0.42)	13.6 (2.5)	(−0.42, 0.59)
FSH (mIU/mL)	51.2 (26.2)	—	—	51.9 (28.5)	(−4.40, 4.29)
LH (mIU/mL)	29.3 (14.9)	—	—	30.3 (15.9)	(−1.32, 3.31)

Abbreviation: WHOQOL-BREF: the World Health Organization's quality of life instrument—short version; CI: confidence interval; FSH: follicle-stimulating hormone; LH: luteinizing hormone.

*P < .05 by paired *t*-test compared with baseline;  **P < .01 by paired *t*-test compared with baseline;  ^∗∗ ∗^ *P* < .001 by paired *t*-test compared with baseline.

**Table 4:** The estimates of regression coefficients and standard errors for modeling outcomes of Pittsburg sleep quality index subscales.

Variable definitions	PSQI subscales^†^											
	Subjective sleep quality		Sleep latency		Sleep duration		Habitual sleep efficiency		Sleep disturbances		Daytime dysfuction	
	Coeff	SE	Coeff	SE	Coeff	SE	Coeff	SE	Coeff	SE	Coeff	SE
MRS at baseline (<16 versus ≥16)^‡^	−0.086	0.158	0.126	0.222	−0.233	0.193	−0.259	0.236	0.179	0.141	0.592*	0.237
Sleep quality at baseline (poor versus very poor)^§^	−0.579**	0.177	0.239	0.248	0.293	0.217	0.108	0.264	0.094	0.158	−0.171	0.266
Mean score improvement at week 1 versus baseline	0.020	0.030	−0.060	0.042	0.030	0.037	0.049	0.045	−0.020	0.027	−0.063	0.045
Mean score improvement at week 4 versus baseline	−0.129***	0.031	−0.106*	0.044	−0.159***	0.038	−0.217***	0.047	−0.121***	0.028	−0.120*	0.047
Body Mass Index (<25 versus ≥25)	0.089	0.189	−0.224	0.266	0.385	0.232	0.528	0.283	−0.401*	0.170	−0.381	0.284
Marital status (married versus others)	−0.027	0.170	0.397	0.239	−0.108	0.209	−0.221	0.254	−0.168	0.153	0.090	0.256
Educational level (college or above versus others)	0.004	0.175	0.191	0.246	−0.114	0.215	−0.026	0.262	−0.006	0.157	0.053	0.263
Age	0.002	0.014	0.022	0.020	−0.025	0.017	−0.007	0.021	0.013	0.013	0.021	0.021

^†^PSQI: the Pittsburg sleep quality index.

^‡^MRS: the menopause rating scale.

^§^Sleep quality is an item of the Pittsburg sleep quality index on a 4-point scale: 0 is very good sleep quality; 1 is fairly good sleep quality; 2 is fairly bad sleep quality; 3 indicates very bad sleep quality.

*indicates *P* < .05;  **indicates *P* < .01;  ***indicates *P* < .001.

**Table 5:** The estimates of regression coefficients and standard errors for modeling outcomes of WHOQOL-BREF Scores.

Variable definitions	WHOQOL-BREF scores^†^							
	Physical		Psychological		Social		Environmental	
	Coeff	SE	Coeff	SE	Coeff	SE	Coeff	SE
MRS at baseline (<16 versus ≥16)^‡^	0.217	0.463	0.336	0.523	0.186	0.600	0.625	0.545
Sleep quality at baseline (poor versus very poor)^§^	0.673	0.519	0.101	0.586	0.420	0.672	0.061	0.610
Improvement at week 1 compared with baseline	0.137	0.088	0.046	0.099	0.051	0.113	0.062	0.103
Improvement at week 4 compared with baseline	0.157	0.092	0.070	0.104	0.012	0.119	0.075	0.108
Body mass index (<25 versus ≥25)	0.082	0.556	−0.008	0.628	0.597	0.720	−0.321	0.654
Marital status (married versus others)	0.136	0.500	0.705	0.565	1.335	0.647*	0.625	0.588
Educational level (college or above versus others)	−0.458	0.515	−0.121	0.582	−0.298	0.666	−0.716	0.605
Age	0.013	0.042	0.023	0.047	−0.040	0.054	0.011	0.049

^†^WHOQOL-BREF: the World Health Organization's quality of life instrument—short version.

^‡^MRS: the menopause rating scale.

^§^Sleep quality is an item of the Pittsburg sleep quality index on a 4-point scale: 0 is very good sleep quality; 1 is fairly good sleep quality; 2 is fairly bad sleep quality; 3 indicates very bad sleep quality.

*indicates *P* < .05;  **indicates *P* < .01;  ***indicates *P* < .001.
